# Unraveling Brain Microcircuits, Dendritic Spines, and Synaptic Processing Using Multiple Complementary Approaches

**DOI:** 10.3389/fphys.2022.831568

**Published:** 2022-02-28

**Authors:** Alberto A. Rasia-Filho

**Affiliations:** ^1^Department of Basic Sciences/Physiology, Graduate Program in Biosciences, Universidade Federal de Ciências da Saúde de Porto Alegre, Porto Alegre, Brazil; ^2^Graduate Program in Neuroscience, Universidade Federal do Rio Grande do Sul, Porto Alegre, Brazil

**Keywords:** brain cytology, neural networks, neural plasticity, neuronal morphology, higher-order processing, synaptic plasticity

## Introduction

**Figure 1:**
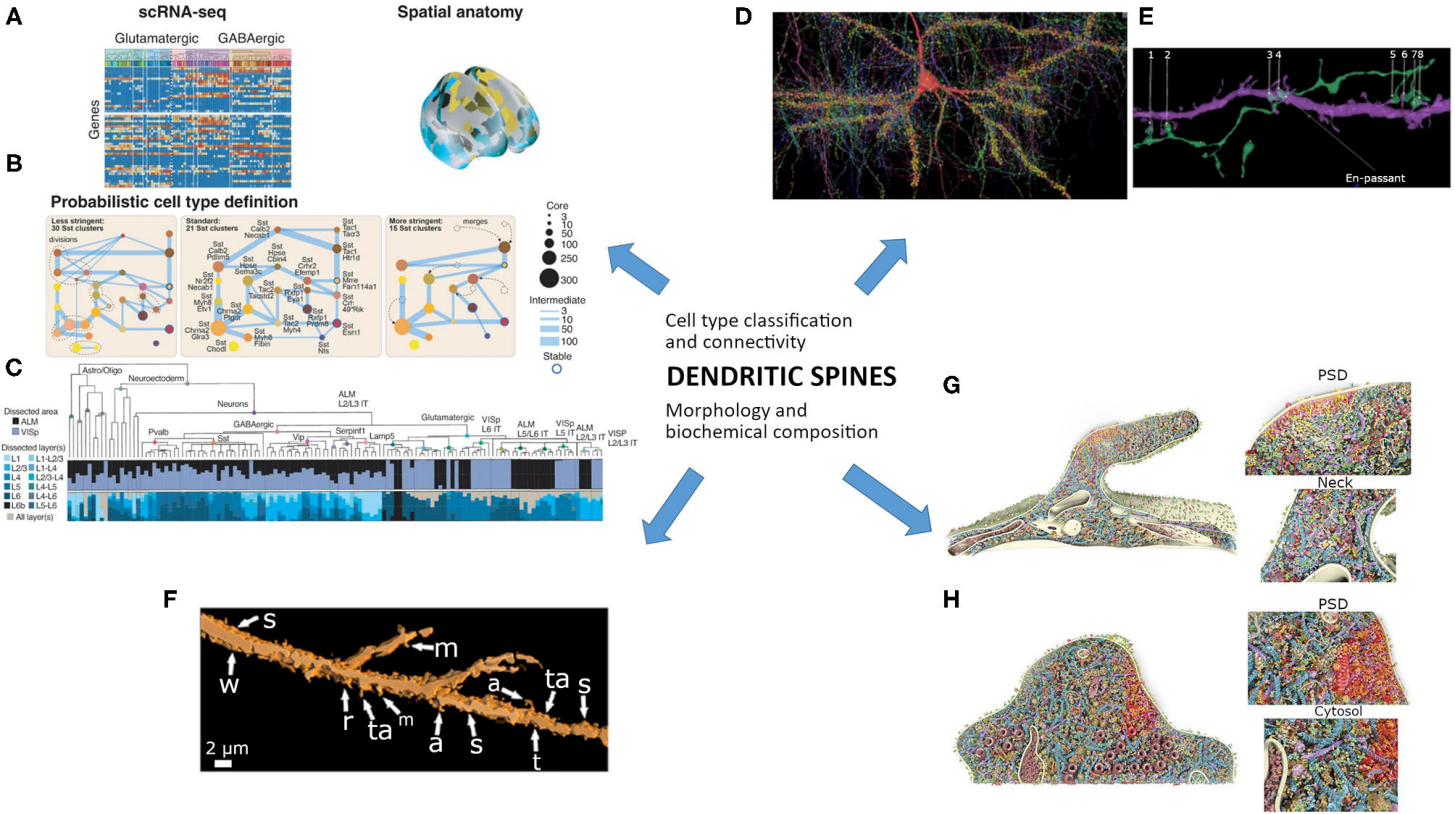
The study of dendritic spines needs to integrate different current fields of knowledge into a coherent vision. Cell type classification and connectivity would be associated with morphology and nanoscale biochemical composition. **(A–C)** The probabilistic definition of a neuronal type needs that: **(A)** “A transcriptome-based cell-type taxonomy is constructed from scRNA-seq data, related epigenomic datasets and neuroanatomy. **(B)** Cell types are initially defined based on transcriptomic signatures in a probabilistic manner with multiresolution clustering and statistical analysis to identify robustness and variability. **(C)** Reproducible gene expression patterns identify hierarchies of putative cell types that are subject to further analyses and validation.” **(A–C)** are reproduced and adapted from Yuste et al. (2020) “A community-based transcriptomics classification and nomenclature of neocortical cell types” (a–c images from Figure 5 were reused without changes), Nat. Neurosci. 23, 1456–1468, doi: 10.1038/s41593-020-0685-8; used under CC BY 4.0 license and copyright. **(D,E)** Image of a petascale reconstruction of human parietal cortex for the study of cells and connectivity. **(D)** “The axonal innervation to a “pyramidal cell (red) is rendered along with all incoming synapses (yellow discs) and presynaptic axons.” **(E)** “Excitatory axon forming 8 synapses onto a spiny dendrite of an excitatory cell. One of these connections is *en passant*, the rest required directed growth of the axon to contact this same dendrite.” Note the axo-spinous contacts (numbered 1, 2, 5–8), which would be further studied by focused ion beam/scanning electron microscopy. **(D,E)** are reproduced and slightly adapted from Shapson-Coe et al. (2021) “A connectomic study of a petascale fragment of human cerebral cortex” (removed letters and changed number size from the original Figure 6), bioRxiv 2021.05.29.446289; doi: 10.1101/2021.05.29.446289; used under aCC-BY-NC-ND 4.0 International license and copyright. **(F)** Morphological features of 3D-reconstructed dendritic spines of a Golgi-impregnated neuron from the human cortical amygdala. Spine shapes include: stubby (s), wide (w), thin (t), mushroom-like (m), ramified (r), transitional (ta), atypical (a) or multiform. Reused and slightly adapted from Vásquez et al. (2018) “Neuronal types of the human cortical amygdaloid nucleus” (Figure 6e), J. Comp. Neurol. 526, 2776–2801. doi: 10.1002/cne.24527; under license # 5223180562839 from Copyright Clearance Center's RightsLink® originally published by John Wiley & Sons, Inc. **(G,H)** 3D model of dendritic spines using large-scale nanoscopy and biochemistry analysis. Multiple constitutive proteins are colored and “shown to scale, with the copy numbers and locations measured in this study and configurations according to literature. For clarity, the highly abundant monomeric actin is not shown. **(G)** View into a mushroom spine. Magnifications into the postsynaptic density (PSD, highlighted with red glow) and neck are depicted. **(H)** View into a stubby spine. Again, a magnification of the PSD is shown and a zoom into the cytosolic region of the spine.” Reused and slight adaptation from Helm et al. (2021) manuscript) after Helm et al. (2021) “A large-scale nanoscopy and biochemistry analysis of postsynaptic dendritic spines” (removed letters in Figure 7), Nat. Neurosci. 24, 1151–1162. doi: 10.1038/s41593-021-00874-w under license # 5223201496130 from Copyright Clearance Center's RightsLink®, originally published by Springer Nature.

Innovative experimental approaches and technological advancements have provided an unprecedented level of detail for the nervous system. New findings advanced our knowledge about the complexity of genetic profiles, neuroanatomical and connectional parcellation of cortical areas, and cytoarchitectonic and synaptic organization in humans compared to other species (DeFelipe, 2011; Vogt, 2015; Bruner et al., 2017; Hodge et al., 2019; Assem et al., 2020; Benavides-Piccione et al., 2020; Eze et al., 2021; Girskis et al., 2021; Viscardi et al., 2021). We can now observe neuronal features on a nanoscale level and envisage possible links for cells and circuits when identifying genes, constitutive proteins, subpopulations of neurons, networks with high-speed actions, higher-ordered mental states, and a multitude of disparate behaviors (Fuzik et al., 2016; Turcotte et al., 2019; Hodge et al., 2020; Close et al., 2021; Demas et al., 2021; Helm et al., 2021). Therefore, we need to integrate different fields of knowledge about dendritic spines into a coherent vision of where this field of research is going using new techniques (Figure 1).

Dendritic spines are specialized postsynaptic compartments (Yuste, 2010; Stewart et al., 2014; Helm et al., 2021) for monosynaptic or, in some cases, multisynaptic inputs (Brusco et al., 2014; Dall'Oglio et al., 2015). Spines are significant in that their morphology appears to change with various inputs and brain disorders (Chidambaram et al., 2019; Baczyńska et al., 2021). Understanding their role in synaptic connectivity is a fruitful approach to elucidating relationships among connectivity of different cellular structures in the brain. However, the study of spines represents a huge challenge when considering neural networks that show region- and cell type-specific characteristics and differences within and among species [Lavenex et al., 2009; Cembrowski and Spruston, 2019; Hodge et al., 2019; Winnubst et al., 2019; Yang et al., 2019; BRAIN Initiative Cell Census Network (BICCN), 2021]. Complementary approaches to the structure-function relationship of the components of an inherently intricate system as the brain are needed. For example, *in vitro* and *in vivo* studies demonstrate the high level of structural and functional complexity of the cerebral cortex (Scheperjans et al., 2008; Rigotti et al., 2013; Vogt, 2015; Glasser et al., 2016; Real et al., 2018; Leopold et al., 2019; Assem et al., 2020; Finkelstein et al., 2021; Foster et al., 2021). In both allocortex and neocortex, morphological heterogeneity within classical cell types can be the rule as occur for the spiny pyramidal neurons (Morishima and Kawaguchi, 2006; Ramaswamy and Markram, 2015; Cembrowski and Spruston, 2019; Benavides-Piccione et al., 2020; Rasia-Filho et al., 2021). Indeed, discrete and continuous variations may coexist and underlie cell-type diversity, forming a “combination of specification through evolutionarily driven and developmentally regulated genetic mechanisms, and refinement of cellular identities through intercellular interactions within the network in which the cells are embedded” [BRAIN Initiative Cell Census Network (BICCN), 2021]. Henceforth, the probabilistic definition of each neuronal type will require single-cell transcriptomic data associated to morphology (Figure 1A; Hodge et al., 2019; Yuste et al., 2020). Dendritic spines may be further studied in specific subpopulations of neurons and circuits to address their functional roles in information processing (Figure 1B).

## Brain Networks, Cellular Connectivity, and the Relevance of Dendritic Spines

Recent approaches advanced the study of brain cells, microcircuits, and connections. The connectomic study of a fragment of the human temporal cortex (1 mm^3^, >5,000 slices cut at ~30 nm), imaged using a high-speed multi-beam scanning electron microscopy (EM) and three-dimensional (3D) reconstruction, exhibited 57,216 cells and ~133 million synapses in a 1.4-petabyte volume (Shapson-Coe et al., 2021). Dense digital reconstruction of a 0.3 mm^3^ cortical circuit containing ~31,000 neurons, ~37 million excitatory and inhibitory synapses, and 55 morphological cell types served to identify hub neurons that could modulate cortical dynamics (Gal et al., 2021). Additional highly multiplexed, high-resolution brain-wide cell-type mapping, and high-throughput spatially resolved transcriptomics approaches can link cell types with connectivity mapping and functional data (Close et al., 2021) for advanced molecular neuroanatomical maps (Ortiz et al., 2021). Some techniques may link functional data with different spatial scales. For example, patch-clamp electrophysiology and single-cell semi-quantitative PCR would identify neuronal subtypes (Fuzik et al., 2016). On the other hand, high-resolution magnetic resonance imaging (MRI) would locate different nuclei in the brain (Saygin et al., 2017 for human amygdala) and help to identify likely borders for each area of interest (e.g., to separate the medial and cortical amygdaloid nuclei, Dall'Oglio et al., 2013; Vásquez et al., 2018). These data are relevant for understanding the complex expression of emotion in different species (Quirk et al., 1995; Zebarjadi et al., 2021; including mice affiliative touch in prosocial interaction, Wu et al., 2021) and what feelings are to humans (Zeki, 2007; Gendron and Barrett, 2009; de Boer et al., 2012; Diano et al., 2017; LeDoux and Brown, 2017; Fogazzi et al., 2020; Šimić et al., 2021).

To process manifold stimuli from external and internal milieux engenders specialization and functional integration of neural areas, cells, and networks (e.g., Rasia-Filho, 2006; Rasia-Filho et al., 2018; Freiwald, 2020; Barnett et al., 2021). Dendritic spine function comprises an important part of this complex scenario (Ramón y Cajal, 1909–1911; Bourne and Harris, 2009; von Bohlen und Halbach, 2009; Yuste, 2010; Spruston et al., 2013; Dall'Oglio et al., 2015; Helm et al., 2021). That is, spines increase the connectivity between neurons and the packing density of synapses without increasing the brain's overall volume (Bourne and Harris, 2009). This feature adds and maximizes the connectivity repertoire governing the shape of dendritic arbors (Wen et al., 2009). Dendritic spines modulate the excitatory synaptic transmission in the brain. The majority of input contacts on dendritic spines are from glutamatergic axon terminals (Yuste, 2013; but see also GABAergic and dopaminergic innervation in Brusco et al., 2014; Kubota et al., 2016; Iino et al., 2020; Kasai et al., 2021). Spines are morphologically diverse, ranging in a *continuum* of number, shape, and size classified according to their head and neck features (Figure 1C). These include: stubby/wide, thin, mushroom, ramified, “atypical” or multiform spines, including “intermediate” shapes, “double” spines, and thorny excrescences, among others (Fiala and Harris, 1999; Arellano et al., 2007; Bourne and Harris, 2007, 2009; Stewart et al., 2014; Fuentealba-Villarroel et al., 2021; see also Ruszczycki et al., 2012; Pchitskaya and Bezprozvanny, 2020). Spine shape involves local actin organization, second messengers, and organelles (e.g., endoplasmic reticulum and ribosomes, Yuste, 2010; Sala and Segal, 2014; Miermans et al., 2017; Okabe, 2020; for mitochondria see Li et al., 2004). This can lead to biochemical compartmentalization and affect the electrical signaling of synapses (Chen and Sabatini, 2012; Tønnesen and Nägerl, 2016; Obashi et al., 2021). The balance between spine number, structure, and function may represent synaptic processing for learning and memory (Bourne and Harris, 2007, 2009) with stimulus-specific features (Knafo et al., 2005) in selective synaptic ensembles (Hayashi-Takagi et al., 2015). Optogenetic manipulation allowed the identification and erasure of specific synaptic memory traces in potentiated spines of the mouse motor cortex (Hayashi-Takagi et al., 2015). This was a remarkable achievement since the functional mapping of single-spine synaptic inputs to the same dendrite can be highly heterogeneous, as revealed by high-resolution two-photon imaging of auditory-evoked NMDA-dependent calcium transients in mouse cortical neurons *in vivo* (Chen et al., 2011).

Dendritic spine dynamics in different neural circuits result from various phenomena. These include phylogenetic, ontogenetic, and epigenetic events (García-López et al., 2010; DeFelipe, 2011; Reza-Zaldivar et al., 2020). Activity-dependent and activity-independent actions promote stabilization, differentiation, and remodeling with enlargement or shrinkage and pruning of spines (Oray et al., 2006; Zancan et al., 2018; Runge et al., 2020; Kasai et al., 2021). Spines can be found relatively isolated or in clusters in the same dendritic segments, as evidenced after 3D image reconstruction of Golgi-impregnated neurons in humans from our laboratory (Reberger et al., 2018; Rasia-Filho et al., 2021) and other approaches using transmission EM (Arellano et al., 2007; Bourne and Harris, 2009; Brusco et al., 2014; Stewart et al., 2014), rapid structured illumination microscopy and enhanced resolution confocal microscopy (for spinules, Zaccard et al., 2020), high-resolution transmission, focused ion beam (FIB) scanning and EM tomography (Rollenhagen et al., 2020), and/or FIB/scanning EM in humans and other animals (Cano-Astorga et al., 2021). Clustered spines can show spike-timing-dependent cooperativity and plasticity (Tazerart et al., 2020). Therefore, synaptic integration made by each spine type can impact cellular activity differently depending upon its location and spatiotemporal processing along proximal to distal dendritic domains (Spruston et al., 2013). In addition, its passive and/or active biophysical properties associated with those of parent dendrites may play a role (Sala and Segal, 2014; Gidon et al., 2020; Obashi et al., 2021). Dendritic spines modulate both stable and/or transitory connections (Oray et al., 2006) and synaptic plasticity using various molecules in variable biochemical pathways for short-term to long-term cellular effects (Sala and Segal, 2014; Chidambaram et al., 2019).

There are many frontiers to explore the structure and integrated function of dendritic spines for synaptic plasticity. The impact of heterogeneous glial cells and the role of the extracellular matrix in tetrapartite synapses need to be addressed (Chelini et al., 2018; Mederos et al., 2018; Tønnesen et al., 2018; Nguyen et al., 2020; Klimczak et al., 2021). The elucidation of the evolutionary reason for the divergence in gene expression patterns in the cerebral cortex, and the features that determine neuronal diversity and specialization in humans are important (Hodge et al., 2019, 2020; Kalmbach et al., 2021). Regarding the latter, some features of human cortical pyramidal neurons include: (1) larger dendritic length and branch complexity than macaque and mice (Mohan et al., 2015; Benavides-Piccione et al., 2020); (2) a class of calcium-mediated graded dendritic action potentials that would classify linearly non-separable inputs (Gidon et al., 2020); and (3) membrane properties that significantly enhance synaptic charge-transfer from dendrites to soma and spike propagation along the axon (Eyal et al., 2016). This indicates that extrapolations on some neuronal features from other species to the human brain have to be done carefully. Human dendritic spines are systematically larger and longer and exist at higher densities than in the mouse cortex (Benavides-Piccione et al., 2020), likely increasing our capacity of synaptic processing and plasticity (DeFelipe, 2011). Human spines, also, show a high diversity of size and shapes (Dall'Oglio et al., 2015; Vásquez et al., 2018; Rasia-Filho et al., 2021). The functional implication of long “silent” spines (Yuste, 2013) and those of convoluted shapes, observed from subcortical to cortical human neurons (Dall'Oglio et al., 2015; Fuentealba-Villarroel et al., 2021), need additional studies. Multiform spines likely indicate the existence of multisynaptic sites for signaling compartmentalization and further computational possibilities within functional microdomains (Chen and Sabatini, 2012; Dall'Oglio et al., 2015; Reberger et al., 2018).

The postsynaptic density (PSD) is a dense area behind the postsynaptic membrane, as seen by EM. It consists of many proteins, including receptors, ion channels, and adhesion proteins, shared with the membrane, cytoskeletal proteins, and scaffolding proteins, all arranged in a hierarchical fashion (Cohen, 2013). Like dendritic spines, PSDs can display morphological alterations with various physiological and behavioral inputs. PSD area can relate to spine head diameter (Arellano et al., 2007) depending on an NMDA receptor-mediated long-term potentiation plasticity (Borczyk et al., 2019). Stubby and mushroom spines show similar average protein copy number and topology for PSD composition identified after summing EM, stimulated emission depletion microscopy, mass spectrometry, fluorescence microscopy, and 3D reconstruction procedures in cultured hippocampal neurons of rats (Helm et al., 2021; Figure 1D). However, proteins related to synaptic strength, spine dynamics, ion channels, endocytosis cofactors, cytoskeletal structure, signaling and trafficking, secretory proteins, and ribosomes are more evident in mushroom spines (Helm et al., 2021). These findings open the possibility to test different spines also in neuropathological conditions (Forrest et al., 2018; Chidambaram et al., 2019; Runge et al., 2020; Baczyńska et al., 2021; Montero-Crespo et al., 2021). From this perspective, dendritic spines are sexually dimorphic and/or affected by gonadal steroids (Woolley and McEwen, 1993; Rasia-Filho et al., 2012; Luine and Frankfurt, 2020), sexual experience and motherhood (Rasia-Filho et al., 2004; Zancan et al., 2018). These phenomena are relevant to sexual differentiation in healthy brain connectivity and as a biological variable in neuropsychiatric research (Joel and McCarthy, 2017; Rubinow and Schmidt, 2019; Arnold, 2020; Hidalgo-Lopez et al., 2021).

Lastly, the human cerebral cortex shows a highly polygenic architecture (Grasby et al., 2020) and ~16 billion neurons (Herculano-Houzel et al., 2014). One cortical pyramidal neuron can form ~30,000 synapses, 90% of them being excitatory (DeFelipe, 2011). From ~100 trillion spines in the human cortex (Kasai et al., 2021), ~99.5% of all spines lie in pyramidal neurons (Kubota et al., 2016; see also Foggetti et al., 2019) for the organization of the ongoing synaptic transmission from multiple neurochemical circuits (Palomero-Gallagher and Zilles, 2019). This complexity is exemplified by the huge spine density and shape variation in a human CA1 pyramidal neuron related to circuits for memory modulation and self-identity (see Figure 9 in Rasia-Filho et al., 2021). On a spine-by-spine basis (Oray et al., 2006), there can be a high degree of synaptic processing arising from spatiotemporal and functional heterogeneity among individual synapses on the same dendrite, between different neurons, and across and between brain regions (Grant and Fransén, 2020). Synaptic diversity and strength are finely adjusted to code information (Grant and Fransén, 2020), enabling coincidence detection (Chabrol et al., 2015) and merging multimodal inputs from parallel pathways (Soltesz and Losonczy, 2018). The integrated synaptic processing and complex plasticity linked to the role of an increasing number of specialized neurons and glia cells within circuits may ultimately lead to the emergence of multiple sensorimotor, cognitive, emotional, abstract, creative and conscious elaborations, visceral reactions, and behavioral displays (for a parallel discussion see Timo-Iaria and Valle, 1995; DeFelipe, 2011; Jezek et al., 2011; Hodge et al., 2019; Freiwald, 2020; Grant and Fransén, 2020; Rasia-Filho et al., 2021).

## Conclusion

Dendritic spines are key elements for innovative research in integrative physiology. Various approaches can expand our knowledge on spines studying them at both network-scale and synapse-scale in the brain. For example, we still do not know the implications of dendritic spines in different subpopulations of neurons for the cytoarchitectonics, rich networks connections, and complex information processing in the insular cortex. This is an interesting cortical area that is strongly activated when “you see the person you are in love with, try to listen to your own heartbeat, suffer from a headache, or crave for a chocolate cookie” (Gogolla, 2017; see also Benarroch, 2019). As mentioned by Mancuso et al. (2014): “Anatomical changes occur on a full range of scales from the trafficking of individual proteins, to alterations in synaptic morphology both individually and on a systems level, to reductions in long-distance connectivity and brain volume.” Dendritic spines relate to all these processes in the brain and with a notable integrative complexity in humans. Unraveling the dynamic role of dendritic spines for synaptic processing is a task that needs multiple complementary approaches. The level of complexity for this endeavor resides in the fact we are looking for representative data that is likely on the astonishing scale of 10^15^ ongoing connections in the human brain.

## Author Contributions

The author confirms being the sole contributor of this work and has approved it for publication.

## Funding

Grants from the Brazilian Agencies CAPES and CNPq (Brazilian Ministry of Science Technology and Innovation RRID), Grant/ Award Numbers: 314352/2020-1, SCR_002876.

## Conflict of Interest

The author declares that the research was conducted in the absence of any commercial or financial relationships that could be construed as a potential conflict of interest.

## Publisher's Note

All claims expressed in this article are solely those of the authors and do not necessarily represent those of their affiliated organizations, or those of the publisher, the editors and the reviewers. Any product that may be evaluated in this article, or claim that may be made by its manufacturer, is not guaranteed or endorsed by the publisher.
